# Severe autoimmune hemolytic anemia; epidemiology, clinical management, outcomes and knowledge gaps

**DOI:** 10.3389/fimmu.2023.1228142

**Published:** 2023-09-18

**Authors:** Femke V. M. Mulder, Dorothea Evers, Masja de Haas, Marjan J. Cruijsen, Sophie J. Bernelot Moens, Wilma Barcellini, Bruno Fattizzo, Josephine M. I. Vos

**Affiliations:** ^1^ Sanquin Research and Landsteiner Laboratory, Translational Immunohematology, Amsterdam UMC, Amsterdam, Netherlands; ^2^ Department of Hematology, Leiden University Medical Center, Leiden, Netherlands; ^3^ Department of Hematology, Radboud University Medical Center, Nijmegen, Netherlands; ^4^ Department of Immunohematology Diagnostics, Sanquin Diagnostic Services, Amsterdam, Netherlands; ^5^ Department of Hematology, Catharina Hospital, Eindhoven, Netherlands; ^6^ Department of Hematology and Amsterdam Institute for Infection and Immunity, Amsterdam UMC, University of Amsterdam, Amsterdam, Netherlands; ^7^ Department of Hematology, Fondazione IRCCS Ca’ Granda Ospedale Maggiore Policlinico, Milan, Italy; ^8^ Department of Oncology and Hemato-Oncology, University of Milan, Milan, Italy

**Keywords:** autoimmune hemolytic anemia, cold agglutinin disease, severe, mortality, management

## Abstract

Autoimmune hemolytic anemia (AIHA) is an acquired hemolytic disorder, mediated by auto-antibodies, and has a variable clinical course ranging from fully compensated low grade hemolysis to severe life-threatening cases. The rarity, heterogeneity and incomplete understanding of severe AIHA complicate the recognition and management of severe cases. In this review, we describe how severe AIHA can be defined and what is currently known of the severity and outcome of AIHA. There are no validated predictors for severe clinical course, but certain risk factors for poor outcomes (hospitalisation, transfusion need and mortality) can aid in recognizing severe cases. Some serological subtypes of AIHA (warm AIHA with complement positive DAT, mixed, atypical) are associated with lower hemoglobin levels, higher transfusion need and mortality. Currently, there is no evidence-based therapeutic approach for severe AIHA. We provide a general approach for the management of severe AIHA patients, incorporating monitoring, supportive measures and therapeutic options based on expert opinion. In cases where steroids fail, there is a lack of rapidly effective therapeutic options. In this era, numerous novel therapies are emerging for AIHA, including novel complement inhibitors, such as sutimlimab. Their potential in severe AIHA is discussed. Future research efforts are needed to gain a clearer picture of severe AIHA and develop prediction models for severe disease course. It is crucial to incorporate not only clinical characteristics but also biomarkers that are associated with pathophysiological differences and severity, to enhance the accuracy of prediction models and facilitate the selection of the optimal therapeutic approach. Future clinical trials should prioritize the inclusion of severe AIHA patients, particularly in the quest for rapidly acting novel agents.

## Introduction

Autoimmune hemolytic anemia (AIHA) is a rare condition, with an incidence of 1-3 per 100,000 per year (1–3). It is characterized by an increased destruction of red blood cells (RBC) due to autoantibodies that takes place extravascularly (spleen and liver) or in rare occasions intravascularly. Generally, the diagnosis is based on markers of hemolysis and a positive direct antiglobulin test (DAT), that detects antibodies and/or complement on the red blood cells of the patient. Various entities are recognized, i.e. warm AIHA (wAIHA), cold AIHA (cAIHA) and mixed or atypical AIHA (4). These subtypes are distinguished by characteristics and effects of the red blood cell reactive autoantibodies, particularly their immunoglobulin class (IgG, IgM, IgA), thermal amplitude and the extent of antibody-mediated complement activation. In wAIHA, autoantibodies are mostly of IgG class and red blood cell destruction typically occurs *via* binding by IgG-Fc receptors on macrophages in the spleen, although some IgG antibodies may also activate complement. Cold agglutinins (CA) are mostly IgM antibodies that bind to the RBC (typically I antigen) at low temperatures, inducing agglutination and complement activation and subsequent red blood cell destruction. The latter occurs mainly in the liver *via* C3b deposition on RBCs. However, in case of terminal complement activation, this may culminate into intravascular hemolysis.

AIHA is classified as either primary or secondary to an underlying disease, including lymphoproliferative disorders, immunodeficiencies, infectious diseases and other auto-immune diseases (5). Primary cAIHA is referred to as cold agglutinin disease (CAD). The CAs are produced by an indolent B-cell lymphoproliferation in the bone marrow. In the most recent revision of the WHO classification, the presence of a B-cell clone and CAs is defined as a separate entity called CAD-Lymphoproliferative disorder (CAD-LPD) (6). If cold agglutinins arise in the context of another underlying disease such as overt malignancy or infection, this is referred to as cold agglutinin syndrome (CAS) (4).

The clinical course of AIHA is highly diverse and AIHA patients vary not only in underlying diseases, but also in factors such as the antibody isotype, site of red cell destruction, involvement of the complement system, and the bone marrow’s compensatory capacity. Given this heterogeneity, predicting the course of the disease in individual cases is challenging. Many AIHA patients solely require outpatient care. However, several case reports and series have illustrated that AIHA can culminate into a fulminant and even fatal clinical course (7–10). These patients may deteriorate rapidly due to massive hemolysis, leading to organ failure, and require repeated blood transfusions and treatment in an intensive care unit (ICU).

In this article, we review the available literature on severe and fatal AIHA, with a focus on definitions, epidemiology including mortality and causes of death, and risk factors associated with unfavorable outcomes. We also summarize available data on treatment and formulate recommendations for the clinical management of fulminant hemolytic episodes. Finally, we point out knowledge gaps and areas for future research.

## Defining severe AIHA

Severe or fulminant AIHA is poorly defined, as described in a recent review on terminology in AIHA (11). Only 2 of 61 identified articles define severe AIHA, both with different hemoglobin (Hb) cut-offs, one extending the definition with need of daily transfusion (12, 13). Importantly however, hemoglobin at onset, while an easily available parameter, can be affected by many factors, such as accessibility to medical care, comorbidities and age. For example, younger patients generally present with lower hemoglobin levels (12, 14), potentially representing a more severe subgroup, but this might also be related to a higher tolerance of severe anemia leading to delayed presentation.

Certain studies categorize by severity of hemolysis, as determined by laboratory parameters of hemolytic activity, instead of outcome measures (15–17). Das et al. and Ray et al. labelled cases as having ‘severe hemolysis’ if all four of the following conditions were met: Hemoglobin <9 g/dL, indirect bilirubin >2 mg/dl, reticulocyte count >2% and LDH >500IU/L. Cases with less than four aberrant values are labelled as ‘moderate hemolysis’. Of note, in this algorithm, a case with a low reticulocyte count might be labelled as having ‘moderate hemolysis’, whilst this may in fact represent patients with inadequate bone marrow compensation, possibly a subgroup with increased risk for severe outcomes.

Recently, the first international consensus group defined severe AIHA as *when the unsupported Hb level falls below 8g/dL and transfusion is required with an interval ≤ 7 days* (4). While the decision for transfusion is multifactorial, at least this definition contains a clinical outcome measure that may capture the complex interplay of various factors related to disease severity (which encompasses not only severity of hemolysis but also transfusion efficacy, bone marrow compensatory capacity, comorbidities, tolerance of hemoglobin level, organ failure et cetera).

Refractory AIHA is a separate specific subgroup, sometimes referred to as severe cases as well. In this review we focus on severe rather than refractory AIHA, although these populations may well overlap.

## AIHA severity and mortality

AIHA severity definitions as per the first consensus meeting or by any other definition have not been validated in clinical studies. Consequently, incidence, characteristics and outcomes of this subgroup have not been established. As a surrogate, data on hemoglobin levels, transfusion burden, hospitalisation, ICU admissions and mortality in the general AIHA population will be discussed.

### Hemoglobin, transfusions and hospital admissions

The Italian GIMEMA group published data on the largest cohort of AIHA patients so far (n=378, follow-up approximately 4 years), consisting of only primary cases (12, 18). In this cohort median hemoglobin at onset was around 7-7.5g/dL, with 63% below 8 g/dL and 27% below 6 g/dL. In a subgroup with more detailed data, health care resource utilisation was analysed (n=190). Of these patients, 54% required blood transfusions at some point, ranging from 1 up to 41 units per year (median 2). Hospital admission was required at least once (up to 8 times) in 64% of the patients during the median follow-up of 4.3 years. Whether transfusions and hospitalizations were due to AIHA, was not specified. The wide range of transfusions and hospitalisations illustrates the clinical heterogeneity of AIHA. Transfusion rates and inpatient hospitalisation rates were significantly higher in wAIHA with IgG plus complement positive DAT, atypical (including IgA mediated and DAT negative forms) and mixed cases. The authors classified 10-15% of cases as ‘severe/ultra-refractory’ AIHA, based on the fact that their hospital stays exceeded 30 days, transfusion exceeded 20 units per year and certain drugs were administered (bortezomib, rituximab, eculizumab). This group mainly consisted of CAD, wAIHA with IgG plus complement positive DAT, mixed and atypical cases.

In CAD, hemoglobin levels are generally higher than in other AIHA subtypes. The largest retrospective multinational cohort of CAD patients (n=232) (19), showed that the median hemoglobin at onset was 9.3 g/dL with 27% of the cohort below 8 g/dL. Up to 47% of patients received at least one RBC transfusion during the median 6 years of follow-up, which is comparable with 51% in the CAD subgroup in the GIMEMA study (median follow-up 4 years) (18).

In both of these cohort studies (18, 19), AIHA-specific transfusions, hospitalisations and complications were not recorded, nor compared to a control group. Two registry-based studies (US and Denmark) analyzed health care utilization by CAD patients in comparison to a control group (matched for age, sex and comorbidity index scores) (20, 21). In both studies, transfusion need within 12 months after disease onset was significantly increased (23.7 vs. 2.1% and 43% vs 1.3% in matched controls). The percentage of patients requiring inpatient hospitalisation at least doubled (36% vs. 15% and 53% vs. 23% in matched controls), and the odds for hospitalisation was 3.9 times higher (20, 21). Similar studies for wAIHA do not exist, but one French population-based study found increased hospitalisation rates for thrombosis (HR 1.9) and infections (HR 4.1) in AIHA (no differentiation between subtypes) compared to matched comparisons from the general population (2).

### ICU admissions

There are no studies reporting on the incidence or frequency of ICU admissions in patients with AIHA. Recently, two French cohorts of AIHA patients admitted to the ICU were published (22, 23). In a single center study, reviewing data over the period 2002-2015, 44 patients were admitted to the ICU department with AIHA (89% secondary AIHA). In 21 of those, ICU admission was primarily because of AIHA, of which all but 2 were admitted for extensive monitoring and transfusion but did not show organ failure on admission. In total, 13/44 patients (30%) died in the ICU, with causes of death being organ ischemia, sepsis and haemorrhage Organ failure upon ICU admission was associated with mortality, however, also 4 (21%) of the patients without any organ failure upon admission died. In a larger multicenter study, performed over the period 2013 to 2020, only ICU admissions primarily for AIHA were included (n=62, 64% secondary AIHA) and compared to a control group of AIHA patients not requiring ICU admission (23). In the AIHA ICU group, 90% received transfusions with a median of 1.5 (range 1.0-2.5) units per day. Multivariate analysis identified hemoglobin and indirect bilirubin at onset as risk factors for ICU admission. During ICU stay, 13% of the AIHA patients died after a median of 3.5 days. Causes of death were cardiac arrest due to refractory AIHA in 5 patients and massive pulmonary embolism in 3. It is important to note that 92% of this cohort displayed inadequate reticulocytosis (Bone Marrow Reticulocytes Index (BMRI) < 121), likely contributing to more severe anemia. However, in multivariate logistic regression analysis, a BMRI <121 was no significant risk factor for ICU admission compared to the non-ICU AIHA group. Of all ICU survivors, 9.3% was readmitted to the ICU for AIHA relapse within 1 month. Nine patients died within one year, of which 6 had ongoing hemolysis at time of death. Causes of death were progression underlying disease (n=3), infection (n=3), pulmonary embolism (n=1), hemorrhagic shock of iatrogenic wound (n=1) and multiorgan failure due to massive hemolysis (n=1).

### Mortality and causes of death

The mortality in the GIMEMA cohort (primary AIHA) was 20% during > 4 years of follow-up, and 11 (3.6% of total cohort) deaths were ascribed to AIHA, with causes of death being infection in 5, myocardial infarction in 1, pulmonary embolism in 1 and multiorgan failure in 4 (12). In the extended cohort (n=378), significant risk factors for fatal outcome were lower hemoglobin at onset (<8 g/dl), presence of Evans syndrome, infections and acute renal failure. No association with thrombotic events and type of AIHA was found (18). The GIMEMA cohort solely consists of primary cases, allowing for analysis of the effect of AIHA itself, rather than the underlying disease. Secondary AIHA is a very heterogenous group with varying proportions of underlying diseases observed among different cohorts and geographic locations (5). Some underlying diseases such as malignancies may have higher mortality rates, regardless of AIHA course, whereas AIHA cases with a temporary trigger such as mycoplasma infection, might never relapse.

In several (predominantly) adult cohorts (8 studies, n=525) with both primary and secondary wAIHA, mortality rates range from 3-20% (24–31). In almost half of the deaths, the cause was unknown or not reported. If mentioned, causes of death were mainly infections (n=21), malignancies and thrombotic events. A clear link with a hemolytic episode is stated in some cases (26, 29), but is generally unknown or not reported. The small cohort sizes, heterogeneity of disease and varying median follow-up times (0-4 years), as well as the lack of a control group limit the interpretation.

The largest multinational (‘primary’) CAD cohort (n=232) analysed survival rates and estimated the five-year mortality at 17%. In this cohort, 11% (3.5% of total cohort) of deaths were ascribed to CAD or its complications (19). Specific causes of death and their correlation with hemolytic episodes were not discussed.

Two recent population-based studies on AIHA mortality compared outcomes to an age and sex matched control group from the general population, and report hazard ratios adjusted for comorbidities (aHR). In a French population-based cohort, the one-year mortality was 17.9% (aHR 2.9) for primary AIHA, 28.4% (aHR 3.5) for secondary AIHA with hematological malignancies and 14.3% (aHR 4.6) for other secondary AIHA (2). The diagnosis of AIHA in this study was based on ICD codes, therefore different subtypes of AIHA (cold, warm, mixed) were not distinguished. A Danish population-based cohort showed similar one-year mortality with slightly higher adjusted hazard ratio’s (aHR), 17.3% (aHR 6.5) for primary AIHA and 30.9% (aHR 4.9) for secondary AIHA. The one-year mortality for CAD/CAS in this cohort was 14.5% (aHR 3.2) (32). Hazard ratios were even higher in the first 100 days after AIHA diagnosis and decreased during follow-up, still being significant for primary AIHA cases 10 years after diagnosis (aHR 1.4), but not for secondary AIHA and CAD. Mortality in AIHA patients is frequently attributed to underlying disease, but both the population-based studies show increased mortality even in primary AIHA (except for primary AIHA under 30 years), compared to a matched control group. In both studies, the diagnosis of AIHA (and distinction of CAD/CAS), as well as defining primary and secondary cases, is solely based on ICD codes in absence of (laboratory) data to confirm diagnosis. Since AIHA diagnostic criteria are complex, diagnosis registration might be incomplete and correlation with associated diseases cannot be confirmed. Moreover, association with comorbidities is highly variable, as AIHA can sometimes be the first symptom of the underlying disease, or underlying malignancies may manifest after AIHA diagnosis (33). Indeed, in one study, underlying hematological malignancy was the most common cause of death in the first year after diagnosis in secondary AIHA, but also increased in primary cases (with adjusted hazard ratio of 10 versus comparators) (32).

The Danish population-based cohort elaborated on causes of death in comparison to the general population (32). Anemia as a cause of death was significantly higher in all AIHA subgroups during total study follow-up (>10 years). 2.8% of primary AIHA cases died of cardiovascular causes within 100 days after diagnosis. Mortality from cardiovascular disease was increased in all AIHA subgroups, and in primary AIHA and CAD, this increased risk persists more than 10 years after diagnosis. This might reflect a more complex interplay of hyperinflammation and (chronic) hemolysis leading to increased thrombotic risk, or side effects from therapies (34). Other significantly increased causes of mortality were infections and bleeding. Increased risk of death by infections can be (partially) attributed to immunosuppressive therapies, which are the cornerstone of AIHA treatment, demonstrating the need for less immunosuppressive therapeutic options. Additionally, AIHA can be secondary to infections or primary immunodeficiency. Increased mortality due to bleeding is difficult to interpret, as no details on type of bleeding are available, but could be related to concomitant ITP (Evans syndrome) or anticoagulation therapies for (prevention of) thrombotic complications. For all causes of death, the time-relation between deaths (i.e., cardiovascular) and hemolytic episodes remains unclear based on the currently available data. Therefore, drawing conclusions on causal mechanisms and high-risk conditions for AIHA patients remains difficult.

## Risk factors for severe clinical course and mortality

Overall, there are no validated prognostic tools to predict the clinical course of AIHA. However, the various cohorts as discussed above identified potential risk factors for severe outcomes, as shown in **Table 1**. In primary AIHA, mortality is associated with Evans syndrome, infections, renal failure and multi-treatment. Low hemoglobin at onset (<8 g/dl) is a significant risk factor for ICU admission, relapse and mortality. WAIHA with complement-positive DAT, mixed AIHA and atypical AIHA are subtypes associated with higher transfusion needs, multi-treatment and hospitalisation. It is worth mentioning that, based on anecdotal evidence and case reports, IgM warm AIHA (atypical AIHA, rare) are generally very severe and difficult to diagnose (often DAT negative), and mainly show a dismal fulminant course (36–38). The same can be said for IgA-only mediated AIHA, which can be easily missed as polyspecific DAT will often be negative. Several fulminant IgA-only cases with severe (intravascular) hemolysis have been reported (39–41).

**Table 1:** Potential risk factors for unfavorable outcome in AIHA.

Risk factor	Outcome	Source
Reticulocytopenia	Life-threatening/fatal	Case series. (8) Expert opinion. (4, 35)
Intravascular hemolysis (hemoglobinuria)	Life-threatening/fatal	Case series. (8) Expert opinion. (4, 35)
High indirect bilirubin at onset	ICU admission	AIHA cohort (23)
Low hemoglobin at onset (<8g/dl)	ICU admission	AIHA cohort (23)
	Relapse/multiple treatment lines	Primary AIHA cohort (12)
	Mortality	Primary AIHA cohort (18)
Serological type: wAIHA (IgG + complement), mixed, atypical.	Need for transfusion	Primary AIHA cohort (18)
	Multiple therapy lines	Primary AIHA cohort (12)
	Hospitalisation	Primary AIHA cohort (18)
Evans syndrome	Mortality	Primary AIHA cohort, case series (8, 12)
Infection	Mortality	Primary AIHA cohort (12)
Renal failure	Mortality	Primary AIHA cohort (12)
Multiple treatment lines	Mortality	Primary AIHA cohort (12)

## Management of severe AIHA

**Figure 1:**
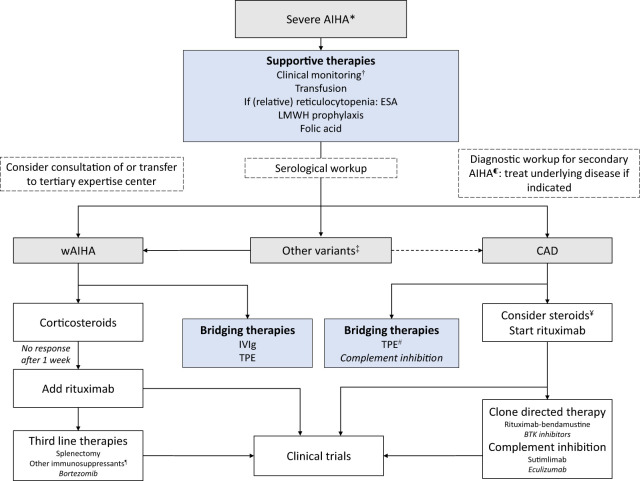
Algorithm for the management of severe AIHA. AIHA = autoimmune hemolytic anemia; ESA = erythropoiesis-stimulating agents; LMWH = low molecular weight heparin; IVIg = Intravenous immunoglobulins; TPE = Therapeutic plasma exchange. *Lower level of evidence in AIHA (see text).* * Typically, unstable hemoglobin <8g/dl and/or hemodynamic instability and/or transfusion interval <7 days. ℂ Underlying diseases including, but not limited to: hematological malignancies, infectious diseases, other auto-immune disease (SLE), primary immunodeficiencies (4). † Daily monitoring of hemoglobin and hemolysis parameters. Consider monitoring in ICU. ‡ IgA AIHA, Mixed AIHA; rare and sometimes aggressive forms, typically recommendations for wAIHA are followed, consultation of expertise center is indicated. ¥ High dose steroids may be effective in severe CAD cases. Start tapering once rituximab has been started and discontinue in <6 months. # Therapeutic plasma exchange in CAD should occur at 37°C (including extracorporeal circuit); exchange plasma for albumin and not donor plasma. ¶ i.e., mycophenolate mofetil, cyclophosphamide, cyclosporin, azathioprine, danazol.

With the exception of one small clinical trial in severe complement mediated AIHA (42), there are no prospective intervention studies specifically focused on the severe AIHA subgroup. All recommendations on how to manage patients presenting with severe AIHA are therefore based on extrapolation of treatment of AIHA in general and expert opinion. Recent consensus on treatment of AIHA, with an emphasis on severe cases, is summarized in **Figure 1** (4, 43, 44).

A full diagnostic work up is warranted, to establish the AIHA subtype and confirm or rule out any underlying cause. If AIHA is secondary, treatment of the underlying disease might be primarily indicated, or can guide the choice for AIHA treatment (45). However, in case treatment of the underlying disease is not required or available, secondary AIHA could be treated as if primary. Especially in severe cases, general AIHA treatment should not be delayed. Additionally, although of great importance, incomplete diagnostic work-up as well should not delay any necessary treatment, including blood transfusions. Expert opinion on diagnostic testing is published elsewhere (4). Consultation of an expertise center is recommended for complex cases with severe AIHA.

A patient with confirmed or suspected severe AIHA should be admitted and monitored including (at least) daily hemoglobin and hemolysis parameters. Unstable hemoglobin, reticulocytopenia, symptomatic anemia, impending organ failure and/or other risk factors as discussed above, should trigger hospitalisation for close monitoring until a stable clinical situation is achieved. There should be a low threshold for ICU admission for intensive monitoring not limited to cases with organ failure.

### Blood transfusion

Similar as for other patients with anemia, the need for blood transfusion in AIHA patients is determined by the physician based not only on hemoglobin levels, but also on clinical parameters as per general guidelines. There is no consensus on a specific trigger for transfusion in AIHA. In wAIHA and to a lesser extent in cAIHA, the identification of possible alloantibodies during pretransfusion work-up can be challenging due to the presence of red blood cell reactive autoantibodies (usually pan-reactive). Additionally required techniques to perform serological analysis are time consuming and might warrant consultation of an expert laboratory. Blood products may be selected that not only are ABO and RhD matched, but also (depending on local facilities) prophylactically matched for Rh phenotype (CcEe) and K to minimize alloantibody formation. In some transfusion guidelines, blood products selected for AIHA patients are always Rh phenotype and K matched (46, 47). In 2/3^rd^ of 115 transfused primary AIHA patients studied, RBC transfusions were effective (in this study, defined as increment of 1 g/dl, stable for 3 days). Although, the efficacy was significantly lower in patients with Hb <6 g/dl compared to Hb 6.1-8.0 g/dl (57% vs 75%), indicating less benefit in more severe cases (12). General hesitance towards transfusion in AIHA patients can be attributed not only to the complexity of pre-transfusion testing but also the fear of adverse reactions because of positive crossmatches. Although this has been reported (48), in general transfusions in AIHA patients are well tolerated as several (small) cohorts found no hemolytic transfusion reactions (49–51). Moreover, the inflamed state of AIHA patients might make them more susceptible to develop alloantibodies, mainly of Rh specificity (52). Given the arguments presented above, despite limited evidence, it is reasonable to minimize the use of transfusions in AIHA patients. However, for vital indications (severe anemia < 6g/dl, cardiovascular risk factors) blood transfusions should not be withheld, and in case of hypoxemia symptoms, administered without delay, even if pre-transfusion work-up is incomplete. Timely communication between clinicians, immunohematology laboratory and transfusion service is essential for appropriate work-up and preventing undesired transfusion delay (52). In case of incompatible blood products, it makes sense to start steroids before the first unit is transfused, if possible, since this may lower the risk of alloantibody formation (53). In cAIHA patients, blood products should be administered with use of a blood warmer. Although only based on expert opinion, the rationale is in line with widely accepted recommendations (4, 46, 54). For adequate monitoring of efficacy and 281 hemolysis stability, regular transfusion (i.e., 1 unit/day) is preferred above multiple units at once (4).

### Therapeutic strategies

In wAIHA in general, predniso(lo)ne (1mg/kg per day) is effective (~80%) and time to response is estimated at 7-25 days (44). High dose intravenous methylprednisolone bolus at initiation of therapy in acute cases is suggested, but solely based on experience in other autoimmune diseases. In CAD, steroids are ineffective except for CAD patients at sustained high doses (0.7-1 mg/kg/day), and may be considered for hemolytic crises, but should not delay more effective treatment options (43). In fact, rituximab (375 mg/m^2^ weekly for 4 weeks or 1000mg fixed dose bi-weekly) is the first choice for CAD and is generally regarded as second choice for wAIHA patients, although time to response is 3-6 weeks. Early administration of rituximab is recommended in severe cases of CAD and wAIHA unresponsive to steroids (i.e., within 1 week), even though immediate effect is not anticipated.

#### Bridging therapies and supportive care

Severe cases might rapidly deteriorate despite multiple blood transfusions, while not (yet) responding to steroids, rituximab or other therapeutic agents. In these situations, clinicians can resort to bridging therapies, such as intravenous immunoglobulins (IVIg) in case of wAIHA or therapeutic plasma exchange (TPE). The response (if response occurs) of both interventions is generally quick (within days), but short-lived. Evidence for both interventions is scarce, as efficacy for IVIg was only 32% in one small study, and use of TPE in AIHA is solely based on case series (55, 56). In case of CAD, TPE should occur at 37 °C and with albumin instead of donor plasma, as colder temperatures and a new complement source could potentially aggravate the disease.

The addition of erythropoiesis-stimulating agents (ESA) such as recombinant erythropoietin (rEPO), may be beneficial based on retrospective data, especially when there is (relative) reticulocytopenia (57). This supportive treatment may be underutilized, as nearly all (92%) ICU-admitted patients in the French cohort had inadequate reticulocytosis, and only 20% received ESA (23).

Thrombosis and infection are known complications in AIHA and among the leading causes of death (58, 59). Even though thrombotic events were not significantly associated with fatal outcome in the largest cohort, AIHA is associated with increased thrombotic risk and cardiovascular mortality (32, 60–62). Predictors for thrombotic risk are not fully clarified, but may be warm and mixed AIHA, and active and intravascular hemolysis (58, 59). Therefore, thrombosis prophylaxis is strongly advised, especially in hospitalized patients with severe anemia and LDH >1.5 times upper limit of normal, and/or additional risk factors for thrombosis (i.e., antiphospholipid syndrome) (4). There are no infectious prophylaxis guidelines for AIHA. Awareness of infection risks is important, and prophylaxis can be considered in individual cases based on local guidelines (63).

#### New therapeutic developments

A proportion of patients deteriorate despite currently available therapies. Indeed, there is a lack of effective, rapidly acting agents to halt the massive hemolytic activity in severe AIHA. Complement inhibitors are emerging in complement-mediated AIHA (typically cold antibody AIHA and, to some extent, wAIHA with complement positive DAT). Several phase II-III trials of C1 inhibition in CAD have shown favourable results (64–66). This had led to the approval of the C1s inhibitor sutimlimab by FDA and EMA for the treatment of CAD (67, 68). However, there currently are no data on sutimlimab in the setting of severe complement-mediated AIHA. A small pilot phase II study on peri-transfusional administration of plasma derived C1 inhibitor in severe complement mediated AIHA showed negative results, despite a significant decrease of C3d deposition on RBCs (42). Studies with various other proximal complement inhibitors are ongoing (69–71). Pegcetacoplan, a C3 inhibitor, has shown effective and is approved for paroxysmal nocturnal hemoglobinuria (PNH). A phase III trial in primary AIHA (both CAD and wAIHA) is ongoing. Preliminary results of an earlier phase II trial indicate a higher efficacy in CAD compared to wAIHA with complement positive DAT, and suggest no effect in wAIHA with complement negative DAT (72). Eculizumab, a terminal C5-inhibitor also approved for PNH, had an effect on hemolysis and decreased transfusion needs in CAD patients in one prospective clinical trial. However the effect was modest, most likely due to ongoing C3-mediated extravascular hemolysis (73). Although limited data are available in severe CAD, a theoretical benefit from C5 inhibition, preventing intravascular hemolysis *via* the membrane attack complex (MAC) route, could be of added value in fulminant cases. This has led to recommendations for using eculizumab in these severe cases (4), but this hypothesis needs further study. Moreover, the overwhelming complement activation in severe cases might ask for higher or more frequent dosing. Therefore, while complement inhibitors are potentially very effective in halting hemolysis in fulminant complement mediated AIHA, their exact efficacy and optimal dosing in this setting remains to be determined.

Not all severe AIHA patients would benefit from complement inhibitors, since hemolysis is not primarily complement driven in the majority of wAIHA. Furthermore, complement inhibition does not reduce the pathogenic antibody production, and would presumably be a life-long treatment in the majority of cases. Indeed, several other agents are explored in clinical trials for AIHA, mainly for relapsed and refractory cases. Some of these agents might have potential in the acute setting, depending on time to response (74).

Phosphoinositide 3-kinases (PI3K) are involved in cell metabolism and survival and delta isoforms are selectively expressed in hematopoietic cells, crucial for B-cell development and proliferation. Parsaclisib, a PI3Kδ inhibitor, achieved a (partial or complete) response in 64% (even 75% in wAIHA subgroup) in a phase II trial (n=12), with hemoglobin levels improving within 2 weeks (75). A phase III trial in wAIHA is currently ongoing (76).

Bruton’s tyrosine kinase (BTK) plays an important role in B-cell activation and Fcγ receptor signalling in macrophages. BTK inhibitors are approved for B-cell lymphoproliferative neoplasms and are increasingly studied in autoimmune disorders. Retrospective data shows ibrutinib (first generation BTK inhibitor) was effective in 90% of cAIHA patients (n=15, 4 CAD, 11 CAS). All 11 transfusion-dependent patients became transfusion-independent of whom 9 within 1 month (77). In a pilot study of ibrutinib in 2 cases with relapsed/refractory primary wAIHA, both cases showed response and were transfusion independent within 2 weeks (78). An open-label phase I-II trial studied rilzabrutinib (second generation BTK inhibitor) in patients with immune thrombocytopenia (ITP), an immune cytopenia similar to AIHA. The BTK inhibitor was effective in 40% of the patients, and a majority showed improved platelet counts within 2 weeks (79). Currently, a phase II trial with ibrutinib is recruiting participants with relapsed/refractory warm or mixed AIHA (80). A phase II trial with zanubrutinib (second generation BTK inhibitor) in CAD has been announced (81).

An alternative approach to reduce antibody mediated hemolysis in wAIHA, is through inhibition of spleen tyrosine kinase (SYK) signaling. The inhibition of SYK-dependent signaling of Fcγ receptors on macrophages and BCR on B cells is thought to reduce phagocytosis and pathogenic antibody production. In a phase II trial in wAIHA, fostamatinib was effective in 46% of wAIHA patients. Responses were seen within 2 weeks of initiation. A large (n=90) placebo controlled phase III trial however, did not meet the predefined primary endpoint (82).

Persisting CD20 negative long-lived autoreactive plasma cells are suggested as a possible explanation for refractoriness to rituximab (CD20 monoclonal antibody) The proteasome inhibitor bortezomib has multiple immunomodulatory effects and is used for its proapoptotic effect on plasma cells. Its potential has been demonstrated in several case reports of relapsed/refractory wAIHA, with median time to response of 2 to 3 weeks (83). One prospective trial in cAIHA was conducted, with an overall response rate of 32% after 3 months in a heavily pre-treated group of cAIHA patients (84). Whether some responses were quicker is not reported. An alternative approach to deplete long-lived autoreactive plasma cells, are monoclonal antibodies targeting CD38 (highly expressed in plasma cells), such as daratumumab. Daratumumab is approved for the treatment of multiple myeloma. Retrospective data suggests a potential role in warm and cold AIHA, mainly described in the context of post allogeneic bone marrow transplantation. The majority of responses occurred within 2 weeks. A phase I trial in relapsed/refractory AIHA is currently recruiting and a phase Ib/II study with isatuximab, an analogue, in wAIHA is currently ongoing (85, 86).

Finally, nipocalimab, an FcRn blocker, has the potential to induce a quick response, as serum IgG autoantibodies decreased within 8 days in a phase II trial in Myasthenia gravis (87). A clinical trial with nipocalimab in wAIHA is currently ongoing (88).

In summary, numerous strategies for the treatment of refractory and relapsed AIHA cases are explored, with varying success rates. The various targets illustrate the diversity of AIHA pathophysiology and the moderate success rates in some studies might represent our incomplete understanding of AIHA pathophysiology and predictors for the most effective strategy in each individual case. It is important to highlight the lack of inclusion and/or (sub)analysis of severe AIHA patients, as this population might require different dosing or preferred targets than a chronic, less severely hemolytic AIHA subgroup.

## Conclusions and knowledge gaps

AIHA is a heterogenous disease, and a substantial subgroup is at risk for hospitalisation, transfusion dependency and complications. The risk for ICU admission is uncertain, but mortality of AIHA patients in the ICU is high. All AIHA patient groups, apart from primary AIHA under 30 years, have increased mortality compared to the general population. Causes of death include cardiovascular disease, infections and anemia itself. Survival of AIHA patients has only modestly improved since 1980 (32) and increased mortality in both primary and secondary cases emphasizes that development of novel therapeutic options for AIHA is still pertinent.

Some patients may deteriorate rapidly with deep anemia due to uncontrolled hemolysis, but data on the incidence and outcomes of severe AIHA is lacking. This knowledge gap impairs the recognition of patients at risk for severe disease course as well as improvement of management strategies. Future research, on the one hand, should focus on validating risk factors for severe AIHA incorporating clinical characteristics but also immunological variables. Development of diagnostic scores ask for large prospective cohort studies incorporating consensus-based definitions of severe AIHA.

A comprehensive understanding of the pathophysiology, clinical course and outcomes of severe AIHA is crucial to identify unmet needs in its management. Although improved protocols for supportive care, such as thrombosis and infection prophylaxis, may be advantageous, the evidence to support such protocols is limited. Transfusion triggers are unclear, and the risk and benefits of transfusion in this setting are also to be elucidated. With new targeted therapeutic options becoming available, treatment algorithms should go beyond differentiating between warm and cold AIHA and consider immunological markers, underlying diseases, and clinical parameters to guide the timing and order of therapeutic agents. Currently, the lack of knowledge on severe AIHA and clinical trials in this subgroup, obstruct the development of evidence-based treatment guidelines for severe cases. Rapidly effective therapies are lacking, and although new therapies emerge, their potential in acute hemolytic crises is unsure. There is a need for prospective clinical trials in the subset of severe AIHA, to evaluate the efficacy and optimal dosing of (novel) therapeutic agents, especially focussing on strategies that may rapidly abrogate hemolytic activity.

## Author contributions

FM and JV wrote the first draft of the manuscript. All authors equally added critical discussions throughout the writing of the manuscript. All authors reviewed and approved the submitted version.
